# The overlooked potential for social factors to improve effectiveness of brain-computer interfaces

**DOI:** 10.3389/fnsys.2015.00070

**Published:** 2015-05-06

**Authors:** Cheryl Ann Sexton

**Affiliations:** Department of Physiology and Pharmacology, Wake Forest University, School of MedicineWinston-Salem, NC, USA

**Keywords:** brain-computer interface (BCI), neurorehabilitatation, social neuroscience, social behavior, augmenting cognition, neural prosthesis

## Introduction

The surge in development of brain-computer interface (BCI) devices is highly focused on the algorithms, mechanics, and neurophysics of their production (Lebedev and Nicolelis, 2006; Lebedev et al., 2011; Opris, 2013). Here I propose that capitalizing on research findings from the field of social neuroscience can enhance training and effectiveness of BCI devices. BCIs are not just about individual brains but also about brains in interaction with other brains. Learning in a social context is more effective than non-social instruction and countless neurophysiology studies have demonstrated that social interaction actually alters physiology, including changes in neuroplasticity and arousal. Importantly, social interaction also consists of emotional responses that have powerful rewarding qualities and incur reciprocal action. Interdisciplinary cooperation between social neuroscience and BCI innovation has been proposed to promote development of more effective BCIs that demonstrate adaptability during interaction (Mattout, 2012). The challenges of BCI illiteracy, or BCI inefficiency, suggests a vital need to consider all possible contributing factors to decrease the failure rate seen in up to a third of users (Vidaurre and Blankertz, 2010). Additionally, it has been suggested that BCI inefficiency can be reduced by addressing flaws in human training approaches, which have been largely neglected (Lotte et al., 2013). Therefore, the social cues and contexts a patient has when BCIs are integrated and employed should not be overlooked for their potential to improve effectiveness.

## Social intelligence

The human brain, like other primate brains, has evolved to be exquisitely tuned to social interactions (Ghazanfar and Santos, 2004). In fact, sociality might have been the primary force driving the evolution of primate intelligence (Jolly, 1966; Humphrey, 1976; Dunbar and Shultz, 2007). Thus, it is no leap to assume that cognitive abilities would be influenced by social interactions (Ybarra et al., 2008). Importantly, cognitive abilities do not exist as abstract mental activities independent of body and world (Barrett and Henzi, 2005). We are born into an environment that consists of others acting and interacting; successfully navigating this social milieu requires the development of many cognitive skills. These essential capacities include memory, learning, decision-making, and behavioral inhibition as well as more complex abilities such as communicating, problem-solving, teaching, mentalizing, and inferring others' mental states (Gariépy et al., 2014).

## Social cues and contexts

Social contexts include observing and participating in exchanges between individuals, and responding to cues integral to social behavior. These cues include complex stimuli that require simultaneous processing of multiple sensory inputs, including olfactory, auditory, tactile, and visual cues. The emotional responses of self and others are also social cues. Whom we are interacting with matters, as well as the connection we feel with them along a continuum ranging from perceived social isolation to bonding and attachment (Cacioppo and Cacioppo, 2012). The mere presentation of cues specific to social interaction, such as language or images of the same species, is enough to cause neurophysiological changes in brain and behavior. In primates, many studies take advantage of the fact that pictures of objects, abstract images, or other species are not as rewarding as pictures of conspecifics (Wilson and Goldman-Rakic, 1994). Even the nature of the stimulus is relevant—we have a preference for biological movement over robotic movement for encoding behavior (Tai et al., 2004) and children learn new behaviors better from humans than from robots or androids (robots that look like humans) (Moriguchi et al., 2010a,b). The special effectiveness of social stimuli was recently shown when superimposing familiar face images onto the P300 Speller character matrix for ERP-based BCI performance increased accuracy and speed in healthy individuals (Kaufmann et al., 2011) and patients (Kaufmann et al., 2013).

## Social neurophysiology

Neurophysiological effects of social context and stimuli are distributed throughout the nervous system. Multiple areas of the brain respond to social conditions as assessed by a variety of methods, including transcranial magnetic stimulation, functional magnetic resonance imaging, electrophysiology, and molecular studies. To comprehensively describe all the neurological findings is not possible here so only a few are highlighted (see for reviews: Beer and Ochsner, 2006; Adolphs, 2009). The anterior cingular cortex (ACC) integrates complex stimuli, empathy, and decision making (Lavin et al., 2013). The striatum is a vital area for social interaction and reward (Báez-Mendoza and Schultz, 2013). In the inferior fronto-parietal areas, the mirror neuron system (discussed further below) is essential for social learning (reviewed in Rizzolatti and Sinigaglia, 2010). Differences in genetic expression are seen when zebra finches, a social songbird, sing to females directly rather than singing undirected song (Jarvis et al., 1998). Social behavior induces neurogenesis in the adult hippocampus in rodents (Gheusi et al., 2009; Lieberwirth and Wang, 2014; Peretto and Paredes, 2014). The early findings that social enrichment improves cognitive performance, neuronal growth, and overall brain mass (reviewed by Rosenzweig, 2007) followed by decades of confirmation, has led to the now standard practice that animals in research programs are provided appropriate social interactions (Guide for the Care and Use of Laboratory Animals, 2011). Conversely, decades of research have shown social stress during early development results in a number of behavioral and neurochemical deficits in multiple brain areas. For example, social isolation during rearing impairs neurogenesis in the dentate gyrus of the mouse hippocampus as revealed by deficits in spatial memory task (Ibi et al., 2008), and cell proliferation, cell survival, and neuronal differentiation are negatively affected by isolation during adulthood in female prairie voles (Lieberwirth and Wang, 2012).

## Cognitive function

Continuing development of BCIs that treat neurological and psychiatric disorders involving cognitive and emotional impairments suggests the need for the most comprehensive techniques to facilitate success. The research reviewed above clearly shows that cognition is changed by the qualities of social exchanges or social cues. For example, executive function can be increased (Ybarra et al., 2008) or reduced (Richeson et al., 2005) depending on the type of social interaction and even the identity of the actor. Where an individual falls along the continuum of social isolation (e.g., neglect, exclusion) to social connection has important consequences for cognitive abilities. In older adults, correlations between perceived isolation and poor cognitive responses have been shown (Tilvis et al., 2004; Wilson et al., 2007; Dickinson et al., 2011). In fact, brain and behavioral responses differ depending on the specific feeling of isolation/connection to the person with whom one is interacting (Cacioppo and Cacioppo, 2012).

## Learning socially

The social environment can arguably be said to be the richest environment for learning the most complex cognitive skills, pointing out the importance of training methods. A special quality about live social interaction is that it acutely primes the induction of novel responses (Gottlieb, 1991). For example, juvenile sparrows will learn the songs of another species when demonstrated by a live tutor that they do not learn from tape recordings (Baptista and Petrinovich, 1984). The use of social reinforcement has been noted to be particularly useful to improve BCI integration (reviewed in Lotte et al., 2013). Even if social feedback is provided by an android, behavioral change is better than when a computer display provides factual feedback (Ham and Midden, 2014). This suggests that when biofeedback systems are used (e.g., EEG, fMRI, MEG), engaging a person who communicates feedback in addition to computer displays could facilitate acceptance and speed of acquisition during training.

An essential learning substrate lies within the mirror neurons found in the cortical areas in humans; these neurons fire when observing or imitating another's behavior, evoking in the recipient the representational state of the observer's action or emotion (Rizzolatti and Craighero, 2004; Rizzolatti and Sinigaglia, 2010). This allows mere observation of an action to increase motor memory encoding (Stefan et al., 2005; Celnik et al., 2006). Methods in stroke rehabilitation based on the mirror neuron system—action observation, motor imagery, and imitation—take advantage of this opportunity to rebuild motor function (Garrison et al., 2010). Similarly, action observation has been proposed to interact with motor training in neurorehabilitation (Pomeroy et al., 2005), suggesting that cognitive training may also be possible by capitalizing on these mechanisms. For example, in a study with older adults, the combination of physical training and action observation together elicited encoding whereas conditions with only physical training or action observation failed. It is therefore suggested that employing a person who demonstrates the same action as the patient during BCI training would facilitate an increase in encoding more than just using imagery.

## Brain-to-brain coupling

Complex joint behaviors such as communication and social coordination depend on synchronous interactions. Interpersonal synchrony promotes a variety of positive outcomes, including affiliation, liking, rapport, and emotional support satisfaction. It is challenging to measure brain activity simultaneously from two people, but studies that examine inter-person variables such as synchrony have found revealing results using neuroimaging methods (summarized by Konvalinka and Roepstorff, 2012). Inter-personal entrainment of behavior between people occurs when engaged in rhythmic behavior, such as finger-tapping (Konvalinka et al., 2010) or chair-rocking (Richardson et al., 2007) resulting in unintentional coordination. Inducing synchronous activity induces brain-to-brain coupling, which might increase the efficiency of partnerships engaged with BCI use. Indeed, it has been suggested that our joint cognition with other minds increases their efficiency as a unit, particularly when in compromised situations (Wegner et al., 1991). Intriguing results with a multiuser BCI video game based on motor imagery showed improved utility, effectiveness, and engagement (Bonnet et al., 2013), suggesting methods using interacting brains would help reduce BCI illiteracy.

## Brain-to-brain transfer

Perhaps the most outstanding example of social interaction effect is the demonstration of brain-to-brain transfer of information via computer. In a recent study, the neural firing pattern code of a rat performing a memory task was transferred to a recipient rat, who then responded correctly more often than when stimulated with a random code or without stimulation (Deadwyler et al., 2013). Further, a study revealed that sensory information from a rat transmitted via computer included bidirectional reward contingency information that changed behavior of both donor and a naïve recipient rat in a location thousands of miles away (Pais-Vieira et al., 2013). Even human brain to rat brain transfer has been achieved (Yoo et al., 2013). These examples suggest that the day when neural signals can be transmitted between individuals is not out of the realm of possibility.

## Recommendations

BCIs used for neurological rehabilitation require progressive practice with feedback and reward (Dobkin, 2007); here I suggest that capitalizing on social factors will result in better outcomes. The social environment provides a context particularly relevant for fostering the development and change of cognition. Given that the presence of social factors facilitates learning suggests that attention should be given to the conditions by which the BCI is integrated. For example, engage a loved one or a trusted facilitator who can supply emotionally rewarding feedback in training and treatment protocols. Use rewards of social stimuli, such as images of people or positive language, to enhance training, and deliver rewards with people instead of only computer displays. Pay attention to the emotional environment to ensure it is conducive for promoting change. Be aware that socially isolated patients will be more sensitive to negative cues and stimuli. Capitalize on the processes of imitation and action observation to stimulate responses of the mirror neuron system; in other words, have the patient and facilitator do the same task. Even if the patient is unable to perform the task, mere observation can stimulate motor neuron responses. Employ synchronization tasks between the facilitator and patient to increase trust and brain coupling. Finally, most revolutionary, use healthy individuals as donors to stimulate the patient via inter-brain coupling with brain-to-brain interfaces. In conclusion, just as it is essential that BCI development and use rely on the accurate use of technical principles, it is vital not to overlook the value of applying findings from social neuroscience in order to maximize the effectiveness of BCI implementation and integration.

### Conflict of interest statement

The author declares that the research was conducted in the absence of any commercial or financial relationships that could be construed as a potential conflict of interest.
